# Biosynthesized Silver Nanoparticles Modulate Inflammation in a Palatine Wound Model

**DOI:** 10.1002/cre2.70213

**Published:** 2025-09-08

**Authors:** Morgana Francisco Machado Guzzatti, Airam Barbosa de Moura, Ligia Milanez Venturini, Laura de Roch Casagrande, Igor Ramos Lima, Camila da Costa, Ellen de Pieri, Lariani Tamires Witt Tietbohl, Paulo Emilio Feuser, Ricardo Andrez Machado‐de‐Ávila, Yaodong Gu, Anand Thirupathi, Paulo Cesar Lock Silveira

**Affiliations:** ^1^ Laboratory of Experimental Physiopathology, Program of postgraduate in Science of Health Universidade do Extremo Sul Catarinense Criciúma Santa Catarina state Brazil; ^2^ Department of Rehabilitation Medicine Ningbo No. 2 Hospital Ningbo Zhejiang China

**Keywords:** green synthesis, inflammation, oxidative stress, palatal wounds, silver nanoparticles

## Abstract

**Objectives:**

This study aimed to compare the effects of silver nanoparticles (AgNPs) synthesized with Curcumin (*Curcuma longa L*.) or Açai (*Euterpe oleracea*) versus a commercial treatment and photobiomodulation in rat palatal wounds.

**Methods:**

In vitro cell viability tests assessed nanoparticle toxicity. The animals were initially anesthetized, and circular lesions were created in the palatine mucosa using a 4 mm/diameter punch. The first treatment session commenced 24 h after the injury and continued daily for 5 days. Twenty‐four hours after the final treatment, the animals were euthanized, and the palatal mucosa tissue was collected for histological and biochemical analyses.

**Results:**

AgNPs‐Cur significantly reduced pro‐inflammatory cytokines and oxidant markers, increased anti‐inflammatory cytokines, the wound contraction rate, and the collagen area, and reduced the inflammatory infiltrate compared to the controls.

**Conclusion:**

The therapies effectively aided inflammation resolution and accelerated tissue repair. This study highlights potential cost‐effective and efficient alternatives for oral and palatal mucosa wound healing, improving upon standard commercial treatments.

## 1 Introduction

Maintaining the health of the oral mucosa is important in preventing infectious diseases. Lesions in the oral mucosa can cause painful ulcerations that impact the quality of life. The level of discomfort that they cause varies among patients, as individual reactions to oral ulcers differ. The size of an ulcer does not necessarily correspond to the degree of discomfort experienced triggered by the lesion. Several treatments have been tested to prevent complications and minimize patient discomfort, but none have been entirely successful. Accelerating wound healing can reduce the risk of infection and lessen discomfort following periodontal surgeries (Sanz et al. 2009).

In a previous study (Thirupathi et al. 2023), it was possible to observe that gold nanoparticles (GNPs) demonstrated anti‐inflammatory and antioxidant effects in a palatal injury model. Based on this, the present study aims to evaluate the capacity of silver nanoparticles (AgNPs) to reduce the inflammatory process, as they are already recognized to have high antimicrobial potential.

AgNPs show significant bioactivity and low cytotoxicity when used alone or in combination with various antibiotics to combat bacterial resistance. Their promising antibacterial properties are due to their ability to disrupt cell membranes, alter genetic material, cause intracellular damage, and induce oxidative stress in bacterial cells. Due to their small size, nanoparticles have a large surface area that enables them to adhere to cell walls, penetrate cells, and disrupt membrane permeability, resulting in the leakage of cell contents (Alsareii et al. 2022; Kambale et al. 2020; Ullah Khan et al. 2018).

However, the physical and chemical methods of nanoparticle synthesis pose greater risks compared to biological approaches (Alsareii et al. 2022). The choice of a solvent medium, an ecologically correct reducing agent, and a nontoxic substance for NPs' stabilization are important aspects to consider during nanoparticle preparation. Plant‐derived NPs have a manufacturing process with steps without toxicity risks. In this way, the plant extract is combined with a metal salt solution for the green synthesis of metal nanoparticles (Akhtar et al. 2013; Balkrishna et al. 2021; Kumar et al. 2013).

Tian (Tian et al. 2007) also point out that AgNPs coated by green synthesis are even smaller, which increases the exposure of reactive and catalytic sites to rapid interactions during inflammation, proliferation, and remodeling. The healing time of the damaged area depends on the size, dose, and morphology of the silver nanoparticles.

Curcumin (*Curcuma longa* L.) is a multi‐faceted molecule, a polyphenol, derived from the plant's rhizome that interacts with multiple molecular targets involved in inflammation. It has been shown to promote early re‐epithelialization, enhance neovascularization, and increase the migration of various cells, such as dermal myofibroblasts, fibroblasts, and macrophages to the wound site. Despite its potent healing properties, curcumin's effectiveness is constrained by its low bioavailability, poor solubility, and rapid metabolism (Ning et al. 2022). To address these issues, nanoparticle‐based drug delivery techniques are the best option to expand the medicinal uses of curcumin, as these materials can enter tissues and organs and translocate to other cells (Mendes et al. 2022), allowing topical delivery of the agent in an extended manner.

The small size and high surface‐to‐volume ratio of nanoparticles allow them to penetrate the skin barrier and enter cells, making them ideal for optimal delivery in topical applications. The slow and sustained release of the encapsulated curcumin helps minimize toxicity, as the maximum concentration of the drug never contacts the skin all at once (Krausz et al. 2015). Açai (*Euterpe oleracea*) has been described as having several health benefits and its use may represent a potential treatment for several disorders. Additionally, is a purple tropical fruit with many antioxidant nutrients and excellent anti‐inflammatory and healing effects on the skin. Furthermore, it contains unsaturated fats (omega‐3, omega‐6, and omega‐9), vitamins and minerals, and immunostimulants (Interdonato et al. 2023).

A recent study showed that açai has remarkable antioxidant activity against superoxide and peroxyl radicals, attributed to orientin, homorietin, vitexin, luteolin, chrysoeriol, and the abundance of quercetin in the plant (Shim et al. 2016). Furthermore, Kang and Kim (Kang and Kim 2018) demonstrated that this fruit has a healing effect on the oral mucosa, which may be associated with its high antioxidant activity. The objective of this study was to assess the cytotoxicity and therapeutic effects of AgNPs synthesized using green methods combined with either Curcumin (*C. longa* L.) or Açai (*E. oleracea*) on palatal wounds in Wistar rats. This was compared to a commercial standard pharmacological treatment (Omcilon) and an electrophysical agent (photobiomodulation).

## 2 Materials and Methods

For the present study, animal experiments were performed following the guidelines of the National Institutes of Health (Bethesda, MD, USA) for the Care and Use of Laboratory Animals and were approved by the Ethics Committee of University under protocol number 81/2022. Additionally, all procedures adhered to the ARRIVE guidelines (Percie du Sert et al. 2020).

### 2.1 Green Synthesis and Characterization of Silver Nanoparticles

Reduction of different extracts was necessary for the synthesis of AgNPs. A stock solution containing *C. longa L*. and/or *E. oleracea* extract (99% curcumin extract from Neon) was prepared in absolute ethanol. AgNPs‐Cur and AgNPs‐Açai were formed by reducing AgNO3 salt with curcumin or açai and stabilized using sodium citrate (Na3C6H5O7). Initially, curcumin and açai were diluted in ethanol (3 mg/mL) and Milli‐Q water (3 mg). To this, 1 mL of the extract solutions was added to 29 mL of deionized water containing 1% sodium citrate. Both mixtures were heated to 90°C and stirred vigorously. Subsequently, a AgNO3 solution (10 mL) was added dropwise under vigorous stirring until the mixtures cooled down. The resulting solutions had a yellowish‐brown color for AgNPs‐Cur and a brown color for AgNPs‐Açai. The concentration of silver in each solution was 0.125 mg/mL.

The ultraviolet–visible spectroscopy (UV–Vis) method with a SpectraMax Plus model was used to characterize the AgNPs solutions. Measurements of the nanoparticles were performed in the visible range (390–700 nm). Transmission electron microscopy (TEM) with a JEM‐1011 (100 kV) was used to assess the size and morphology of the nanoparticles. A drop of the nano‐particle solution was placed on a 300‐mesh copper grid covered with a thin carbon layer, dried at room temperature for 24 h, and then imaged. Dynamic light scattering (DLS) and zeta potential measurements were performed using a Zetasizer Nano ZS to determine the hydrodynamic size and surface charge at pH 7.4 (25°C). The nanoparticle solutions were placed in a folded capillary cell, and all analyses were conducted in triplicate to obtain the mean and standard deviation (SD). The stability of the solutions was assessed using UV–Vis and Zetasizer spectroscopy.

#### 2.1.1 Cell Culture of NIH3T3

The NIH3T3 immortalized murine fibroblast cell line (Cell Bank of Rio de Janeiro, RJ, Brazil) was utilized for cell culture assays. These cells were cultured in 25 cm² plastic flasks containing DMEM medium, which was supplemented with 1% penicillin‐streptomycin (10 U.L/mL) and 10% heat‐inactivated fetal bovine serum (complete DMEM). The cells were incubated in a humidified environment with 5% CO_2_ at 37°C to support growth and adhesion. The medium was changed every other day, with adjustments as needed, until the cells reached approximately 80% confluence, suitable for in vitro experiments.

Once the desired confluence was achieved, the cells were detached by trypsinization. DMEM was removed from the flask, and 4 mL of trypsin was added for 5 min or until the cells detached. Then, 4 mL of DMEM was added to neutralize the trypsin. The total number of cells was counted using a Neubauer chamber. The cells were then diluted in complete DMEM to the desired concentration, with 500 µL/well added to a 24‐well plate to achieve a final concentration of 1 × 10^5^ cells/well. The cells were incubated for 24 h in a humidified incubator with 5% CO_2_ at 37°C to allow for cell adherence before conducting the assays.

Following the adherence period, the complete DMEM medium was removed from all wells, and NIH3T3 cells were treated with AgNPs‐Cur and AgNPs‐Açai at concentrations of 1%, 5%, 10%, and 20%, diluted in DMEM. The plate was incubated for 24 h in a humidified oven with 5% CO2 at a temperature of 37°C. After 24 h, the treatments were removed, and the reagent (3‐(4,5‐Dimethylthiazol‐2‐yl)‐2,5‐diphenyltetrazolium [MTT]) was added to perform the cell viability assay.

#### 2.1.2 MTT Assay for Cellular Viability

This method aims to assess cell viability through the reduction of MTT, which is water‐soluble and yellow in its oxidized state. When reduced, MTT transforms into formazan crystals, which are purple and insoluble in water. This reduction process occurs within the mitochondria, meaning that cell viability is directly correlated with the amount of formazan produced.

After treatment of the cells for 24 h, a solution of 0.5 mg/mL MTT in PBS was prepared. The treatments were removed from the wells, and 100 µL of the MTT solution was added to each well, followed by a 3‐h incubation (5% CO2 at 37°C) to allow formazan crystals to form. Post‐incubation, the MTT solution was discarded, and 100 µL of isopropyl alcohol was added to each well to dissolve the formazan crystals. The plate's absorbance was then measured at a wavelength of 570 nm using a plate reader.

Cell viability was determined by comparing the absorbance values of the test groups (AgNPs‐Cur and AgNPs‐Açai) to the control group, which consisted of viable cells only. The results were expressed as a percentage of cell viability, with the control group representing 100% live cells. These calculations provided the percentage of viable cells in each group, allowing for the evaluation of the proposed molecule's toxicity.

### 2.2 Experimental Design

#### 2.2.1 Animals

Sixty Wistar rats (weighing 200–300 g and aged 60 days) were utilized for this study. The animals were housed in specific cages, maintained at a controlled room temperature of 20°C–22°C, with a 12/12‐h light‐dark cycle, and were provided with a standard rodent diet and water ad libitum. The rats were randomly assigned to the following five groups (*n* = 12 each):

A. Palatal Wound (PW)—no local or systemic treatment.
B. PW + Photobiomodulation (PBM)—treated with a 660 nm laser at 2 J.
C. PW + Omcilon—treated with standard Omcilon.
D. PW + AgNPs‐Cur—treated with silver nanoparticles reduced with curcumin (0.125 mg/mL).
E. PW + AgNPs‐Açai—treated with silver nanoparticles reduced with açai (0.125 mg/mL).

#### 2.2.2 Wound Preparation

The animals were anesthetized by an intraperitoneal injection of ketamine (100 mg/kg) and xylazine (0.5 mg/kg) and kept under anesthesia. The palatal injury model was induced as described by Thirupathi et al. (Thirupathi et al. 2023). Each rat was stabilized, and the mouth was held open with a retractor. A 4 mm diameter stainless‐steel dermatological punch was used to remove the palatine mucosa near the molar teeth. The circular incision excised the entire palatal mucosa, exposing the periosteum within the same diameter. After controlling the bleeding, the exposed area was left untreated to allow for healing by secondary intention.

#### 2.2.3 Treatment

Twenty‐four hours after the injury was induced, the animals in groups B, C, D, and E were sedated with 4% isoflurane and received their respective treatments. Group B was subjected to the first session of photobiomodulation using an LBP AlGaInP laser with continuous emission (wavelength 660 nm; peak power 30 mW; beam size 0.10 cm²; 2 J) from Laserpulse‐Ibramed (São Paulo, Brazil). The irradiation was applied to five points around the wound, with the laser pen held perpendicular to the palate at a distance of 0.5 cm from each point, following the protocol described by Mendes et al. (Mendes et al. 2020). Group C received a topical treatment of Omcilon (50 µL) (1 mg/g triamcinolone acetonide) applied to the palatal wounds using a disposable microbrush applicator. Groups D and E were treated with AgNPs‐Cur or AgNPs‐Açai (50 µL hydrogel, dose of 0.125 mg/mL, and mean size 20 nm), also applied to the wounds using a disposable microbrush. The dose was chosen based on previous research, where 0.125 mg/mL was shown to be a dose with therapeutic effects and low cytotoxicity (Hamed et al. 2024; Chinnasamy et al. 2021). These treatments were administered for five consecutive days.

#### 2.2.4 Macroscopic Analysis and Inflammatory Score

The macroscopic evaluation of the wound was based on a visual scoring system for inflammation levels, following a protocol adapted from Ribeiro et al. (Ribeiro et al. 2008). The inflammation score included the following parameters:

1. No change.
2. Irritation.
3. Shallow ulcer (1–2 mm) with clean edges.
4. Shallow ulcer (1–2 mm) with necrotic edges.
5. Shallow ulcer (3–4 mm) with clean edges.
6. Shallow ulcer (3–4 mm) with necrotic edges.
7. Deep ulcer (1–2 mm) with clean edges.
8. Deep ulcer (1–2 mm) with necrotic edges.
9. Deep ulcer (3–4 mm) with clean edges.
10. Deep ulcer (3–4 mm) with necrotic edges.
11. Necrotic tissue with increased margins.

### 2.3 Euthanasia

Five days following injury induction and daily treatment applications, the animals were anesthetized with 4% isoflurane and euthanized by decapitation. The injured palatal mucosa, along with the surrounding margin, was excised using a 5 mm diameter stainless‐steel dermatological punch. The tissue samples were then placed in individual flasks for subsequent biochemical and histopathological analyses.

### 2.4 Wound Size Evaluation

The photographic method serves as a precise alternative for measuring wound areas and is suitable for clean, contaminated, or otherwise affected wounds. Digital images of the wounds, taken at a resolution of 3264 × 2448 pixels, were analyzed using ImageJ 1.51 software. These images were captured on days 0 and 6 to visually monitor the healing progress and measure the wound size (area in mm²) by calculating the percentage change in wound areas during this period. A single researcher conducted three measurements for each wound, using the average value for accuracy.

### 2.5 Histological Analysis

Tissue samples from the soft palate (*n* = 5 animals per group) were immersed in a 10% paraformaldehyde (PFA) solution in 0.1 M phosphate buffer (pH 7.4) for 24 h. Following dehydration and bleaching, the samples were embedded in paraffin and sectioned into 5 μm thick slices. Hematoxylin and eosin (H&E) staining was utilized to evaluate the inflammatory infiltrate, whereas Gomori's trichrome staining was used to assess and quantify collagenesis. The sections were examined under an optical microscope (Eclipse 50i, Nikon, Melville, NY, USA) at ×600 magnification. Images were captured using a Nikon camera (Sight DS‐5M‐L1, Melville, NY, USA) and analyzed using NIH ImageJ 1.36b software (NIH, Bethesda, MD, USA). Inflammatory infiltrate counts were conducted using the “Cell Counter” plugin, focusing on the nuclear staining of inflammatory cells. For collagenesis quantification, the “Colour Deconvolution” plugin was used, which measures the percentage of blue color in the image's total area.

### 2.6 Determination of Inflammatory Biomarkers

The samples were prepared, and the plate was sensitized for subsequent incubation with the antibody. Cytokines (TNF‐α, IL1‐β, IL4, and TGF‐β) were measured using the enzyme‐linked immunosorbent assay (Duoset ELISA) capture method (R&D Systems Inc., Minneapolis, USA).

### 2.7 Biochemical Analysis

#### 2.7.1 Intracellular Determination of Oxidative Stress Parameters

The production of hydroperoxides was assessed by measuring the intracellular formation of 2',7'‐dichlorofluorescein (DCFH) from the oxidation of 2',7'‐dichlorodihydrofluorescein diacetate (DCFHDA) by reactive oxygen species (ROS), following the modified method of (Dong et al. 2010). Nitric oxide (NO) production was evaluated spectrophotometrically by measuring the stable metabolite nitrite. Nitrite content was quantified using a standard curve ranging from 0 to 100 nM, based on sodium nitrite (NaNO_2_), and expressed as μmol nitrite/mg protein (Chae 2004).

#### 2.7.2 Antioxidant Defense Assessment

Glutathione (GSH) levels were measured according to the modified method of Hissin and Hilf (Hissin and Hilf 1976). Following protein precipitation with 1 mL of 10% trichloroacetic acid in the palatal mucosa homogenate, an 800 mM phosphate buffer (pH 7.4) and 500 μm DTNB were added to the sample. Absorbance was read at 412 nm after 10 min. GSH levels were calculated using a reduced glutathione standard curve.

#### 2.7.3 Determination of Protein

Protein content in the palatal mucosa homogenate was determined using the method of Lowry and Rosebrough (Lowry et al. 1951), with bovine serum albumin as the standard. Phosphomolybdic phosphotungstic reagent (Folin phenol) was added to bind to the protein, and absorbance was measured at 750 nm.

### 2.8 Statistical Analysis

Macroscopic data were analyzed using IBM SPSS Statistics software version 21.0. Quantitative variables were reported as mean and standard deviation for normally distributed data, and minimum and maximum values (Callegari‐Jacques 2009). Statistical tests were conducted with a significance level of *α*  = 0.05% and 95% confidence. Data normality was assessed using the Shapiro–Wilk test (Field 2009).

For dichotomous qualitative variables, mean comparisons of quantitative variables were performed using the Mann–Whitney *U* test for non‐normally distributed data. For polyatomic qualitative variables, mean comparisons were conducted using the Kruskal–Wallis *H* test, followed by the post hoc Dunn test when statistical significance was found (Vieira 2010). Associations between qualitative variables were examined using the Likelihood Ratio test, followed by residual analysis upon observing statistical significance (Zar 2010).

Other biochemical and molecular data were presented as mean and mean standard error, analyzed by two‐way ANOVA, followed by the post hoc Tukey test. The significance level for statistical tests was set at *p* < 0.05. SPSS version 17.0 was used for statistical analysis.

## 3 Results

### 3.1 AgNPs Characterization

**Figure 1:**
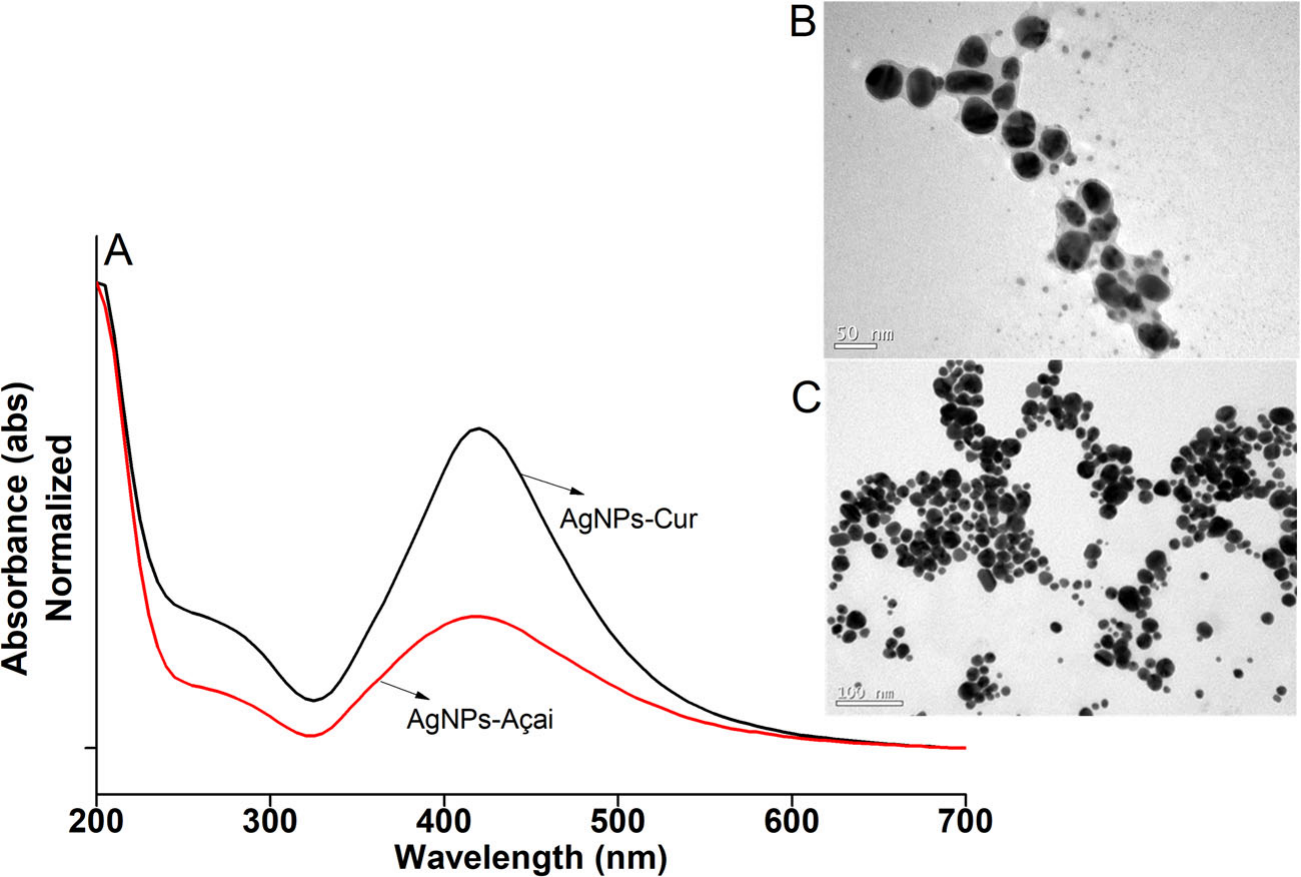
UV–vis analysis (A) and TEM images of AgNPs‐Cur (B) and AgNPs‐Açai (C). *Source:* The author.

Figure 1A shows the wavelength (UV‐Vis) of both AgNPs. AgNps‐Cur and AgNps‐Açai presented the maximum wavelength at 411 nm and 408 nm, respectively (Ashraf et al. 2016). The morphology and size distribution of the particles were examined by Transmission Electron Microscopy (TEM), as can be seen in Figure 1B. Both AgNps synthesized with curcumin or açai present a near‐spherical morphology and a size range between 10 and 40 nm. Particle size distribution (PSD) and surface charge were measured using DLS, as shown in Table 1. The PSD of the AgNps‐Cur (39 nm ± 4) and AgNps‐Açai (32 nm ± 4) was consistent with the data obtained by TEM. The zeta potential of the different nanoparticles was greater than −20 mV (pH 7), indicating good stability. Analysis with a UV–Vis spectrophotometer confirmed the formation of different AgNPs with Curcumin and Açaí. Nanoparticles synthesized with different extracts showed no significant difference (ANOVA *p* < 0.5) in the maximum wavelength. The observed wavelengths corroborate the formation of metallic nanoparticles.

**Table 1:** Average size distribution and zeta potential of different nanoparticles using DLS analysis.

Samples	PSD (nm)	Zeta potential (mV)	Maximum wavelength
AgNPs‐Cur	39 ± 4	−22 ± 3	419 nm
AgNPs‐Açai	34 ± 2	−28 ± 3	416 nm

*Note:* Mean ± standard deviation of three determinations.

*Source:* The author.

### 3.2 Effects of Treatment With AgNPs‐Cur and AgNPs‐Açai on Cell Viability in Fibroblasts

NIH3T3 fibroblasts were exposed to four different concentrations of AgNPs‐Cur and AgNPs‐Açai. The concentrations evaluated were 1%, 5%, 10%, and 20%, with an exposure period of 24 h.

**Figure 2:**
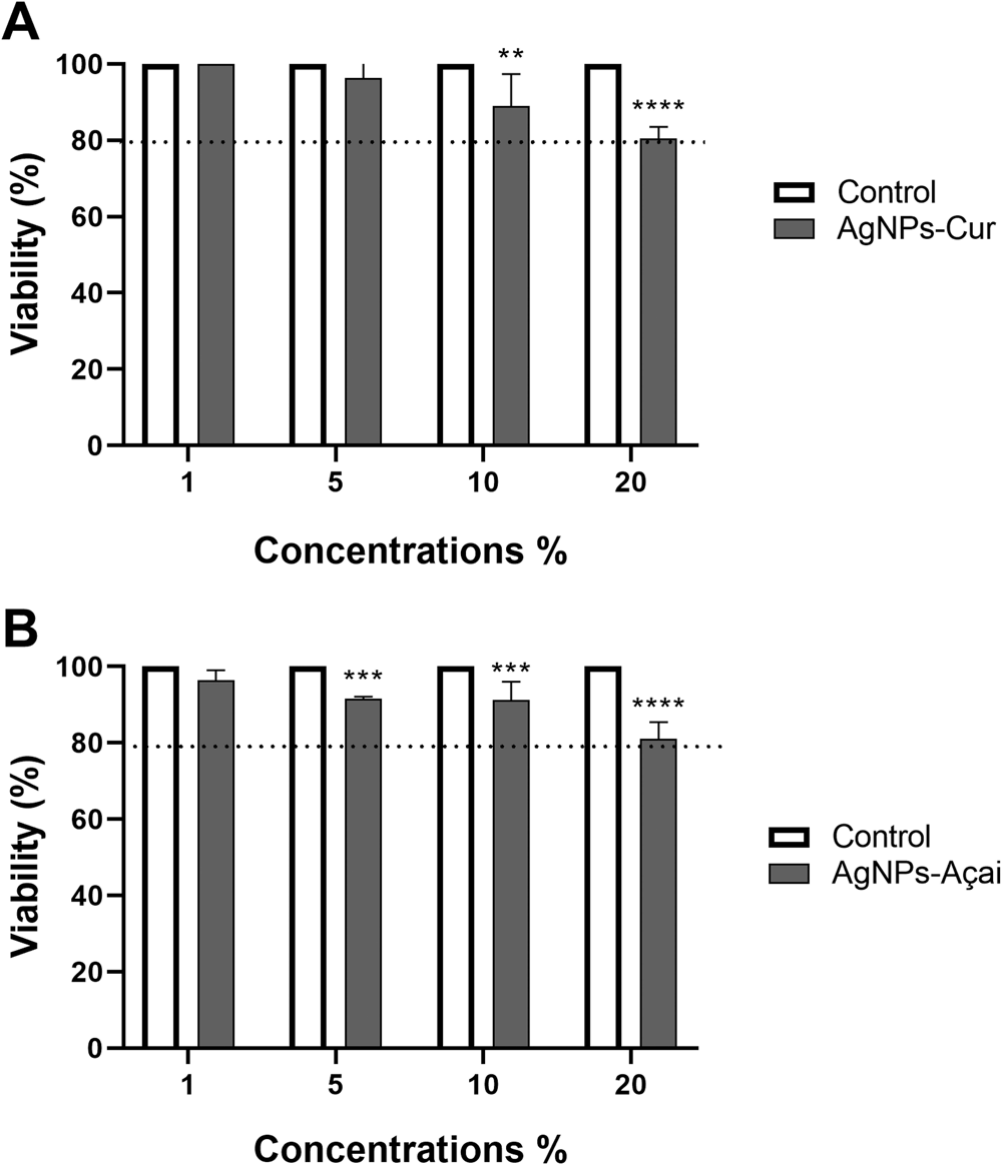
In vitro toxicity of AgNPs‐Cur (A) and AgNPs‐Açai (B) at different doses, evaluated in NIH3T3 fibroblasts over 24 h. The results are presented as mean ± standard error of the mean (SEM), where **p < 0.01, compared to the control; ***p < 0.001 compared to the control; and ****p < 0.0001 compared to the control (two‐way ANOVA, followed by Tukey's post hoc test). *Source:* The author.

As illustrated in Figure 2A, for AgNPs‐Cur, concentrations of 10% and 20% resulted in significant reductions in cell viability compared to the control, but only at 20% did viability approach 80%.

Regarding AgNPs‐Açai, as shown in Figure 2B, concentrations of 5%, 10%, and 20% showed significant reductions compared to the control, but never below 80%.

It is important to highlight that, in both treatments, the 20% concentration was the one with the lowest cell viability, being the most cytotoxic for NIH3T3 cells.

3.3 Macroscopic analysis and inflammatory score in Table 2, the PW + AgNPs‐Cur and PW + AgNPs‐Açai groups did not show a significant difference between them on analyzing the macroscopic perception of the inflammatory condition.

**Table 2:** Macroscopic analysis and inflammatory score.

AgNPs	*n*	M ± SD	Minimum	Maximum	*p* value
Palatal Wound (PW)	12	2.50 ± 0,67	1	3	
PW + PBM	10	5.70 ± 1.76#	2	8	0.001
PW + Omcilon	10	4.80 ± 2.09	3	9	
PW + AgNPs‐Cur	10	4.40 ± 1.50	2	6	
PW + AgNPs‐Açai	12	3.58 ± 1.88	2	8	

*Source:* The author.

^#^ *p* < 0.001 versus PW and PW + Omcilon groups.

However, the PW + PBM group showed a significant difference when compared to the PW and PW + Omcilon groups.

### 3.3 Effects of Treatment With PBM, Omcilon, AgNPs‐Cur, and AgNPs‐Açai on Wound Contraction in a Rat Model of Palatal Wound

**Figure 3:**
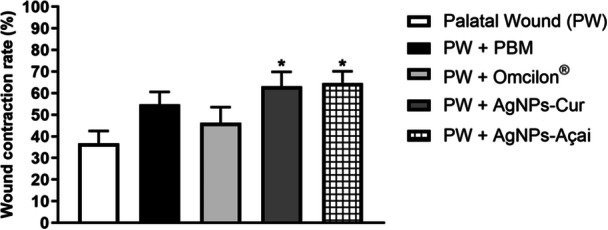
Effects of treatment with PBM, Omcilon, AgNPs‐Cur, and AgNPs‐Açai on the wound contraction rate in %. Data are presented as mean + SEM, where *p < 0.05 versus the palatal wound group (PW) (one‐way ANOVA, followed by Tukey's post hoc test). *Source:* The author.

Figure 3 shows the wound reduction rate in %. A significant increase (*p* < 0.05) in wound reduction was observed in both the group treated with PW + AgNPs‐Cur and the group treated with PW + AgNPs‐Açai compared to the PW group.

### 3.4 Effects of Treatment With PBM, Omcilon, AgNPs‐Cur and AgNPs‐Açai on Histological Evaluation in a Rat Model of Palatal Wound

**Figure 4:**
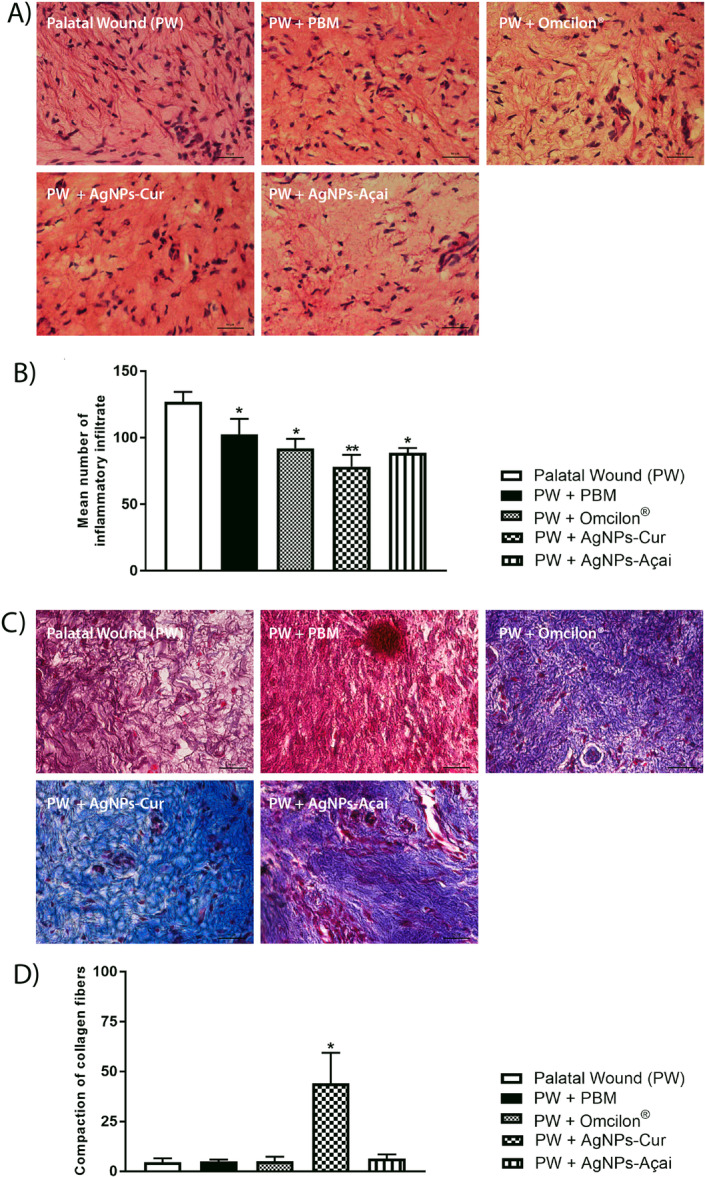
Effects of treatment with PBM, Omcilon, AgNPs‐Cur, and AgNPs‐Açai on histological evaluation, where (A) staining with H&E; (B) mean count of infiltrated inflammatory cells; (C) staining with the Gomori trichrome method; and (D) density of collagen fibers. The results are presented as mean ± standard error of the mean (SEM), with significance indicated by *p < 0.05 compared to the palatal wound group (PW) and **p < 0.01 compared to the palatal wound group (PW) (one‐dimensional ANOVA, followed by Tukey's post hoc test). *Source:* The author.

Figure 4A displays representative images of histological sections of the palatal mucosa, stained with H&E. Figure 4B shows the quantification of the average number of infiltrated inflammatory cells, showing a significant reduction (*p* < 0.05) in all groups compared to the control, with the PW + AgNPs‐Cur group showing an even greater significance (*p* < 0.05) 0.01).

Figure 4C shows representative images of histological sections of the palatal mucosa, stained with the Gomori trichrome method to visualize collagen. Figure 4D shows a significant increase in the collagen area exclusively in the PW + AgNPs‐Cur group compared to the PW group (*p* < 0.05).

### 3.5 Effects of Treatment With PBM, Omcilon, AgNPs‐Cur and AgNPs‐Açai on the Levels of TNF‐α, IL‐1β, IL‐4, and TGF‐β Cytokines in a Rat Model of Palatal Wound

**Figure 5:**
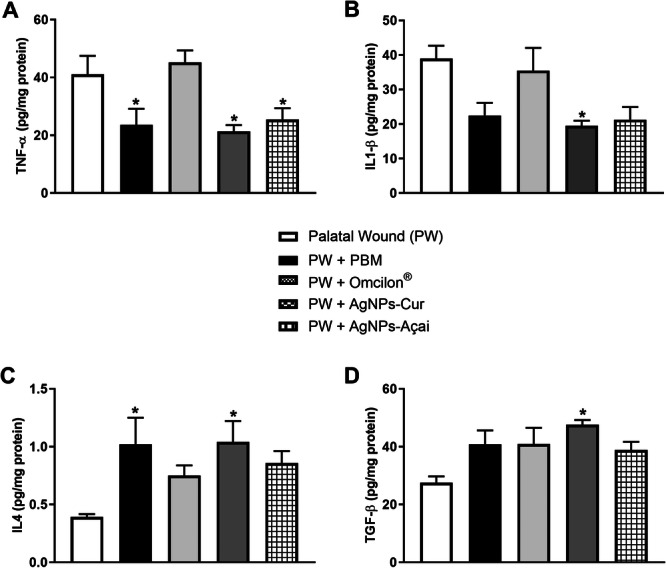
Effects of treatment with PBM, Omcilon, AgNPs‐Cur, and AgNPs‐Açai on the levels of pro and anti‐inflammatory cytokines. In (A) TNF‐α, (B) IL‐1β, (C) IL‐4, and (D) TGF‐β. Data are presented as mean + SEM, where *p < 0.05 versus the palatal wound group (PW) (one‐way ANOVA, followed by Tukey's post hoc test). *Source:* The author.

Figure 5 illustrates the protein levels of the cytokines TNF‐α, IL‐1β, IL‐4, and TGF‐β. With regard to pro‐inflammatory cytokines, the evaluation of TNF‐α levels (Figure 5A) revealed a significant decrease in the PW + PBM, PW + AgNPs‐Cur, and PW + AgNPs‐Açai groups when compared to the PW group (*p* < 0.05). Figure 5B demonstrates a significant reduction in IL‐1β levels in the PW + AgNPs‐Cur group compared to the PW group (*p* < 0.05).

For anti‐inflammatory cytokines, Figure 5C indicates that IL‐4 showed a significant increase in the PW + PBM and PW + AgNPs‐Cur groups compared to the PW group (*p* < 0.05). Regarding TGF‐β levels (Figure 5D), a significant increase (*p* < 0.05) was observed only in the PW + AgNPs‐Cur group compared to the PW group.

### 3.6 Effects of Treatment With PBM, Omcilon, AgNPs‐Cur, and AgNPs‐Açai on Levels of Oxidants and Antioxidant Agents in a Rat Model of Palatal Wound

**Figure 6:**
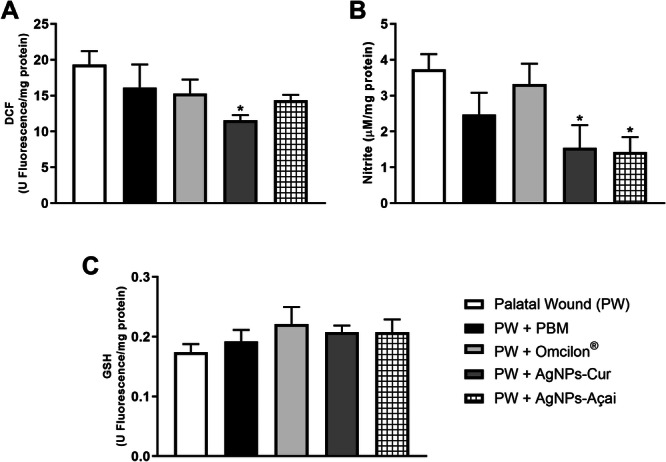
Effects of treatment with PBM, Omcilon, AgNPs‐Cur and AgNPs‐Açai on levels of oxidants and antioxidants. In (A) DCF, (B) nitrite and (C) GSH. Data are presented as mean + SEM, where *p < 0.05 versus the palatal wound group (PW) (one‐way ANOVA, followed by Tukey's post hoc test). *Source:* The author.

For the analysis of oxidative parameters, DCF and nitrite levels were measured, with GSH being an antioxidant indicator. In Figure 6A, a reduction in DCF levels was observed exclusively in the PW + AgNPs‐Cur group compared to the PW group (*p* < 0.05). As for nitrite levels (Figure 6B), both the PW + AgNPs‐Cur and PW + AgNPs‐Açai groups showed a significant decrease (*p* < 0.05) in relation to the control. However, GSH levels did not show significant variation between different groups.

## 4 Discussion

The integrity of the oral mucosa plays a crucial role in protecting against external infections (Chaushu et al. 2020). Oral ulcers are common lesions of the oral mucosa, often presenting similar clinical characteristics, although their causes and mechanisms may vary. Clinical practice and research have advanced the understanding of wound healing processes, which has allowed healthcare professionals to improve treatment and manufacturers to develop new types of dressings (Pallaske et al. 2018).

Nanoparticles used for drug delivery offer significant technological benefits, including high stability, efficient transport capacity, and versatility in administration, whether orally or by inhalation. Alsareii and Manaa Alamri (Alsareii et al. 2022) highlight that AgNPs demonstrate excellent properties, such as electrical conductivity, chemical stability, and catalytic and antibacterial characteristics, in addition to cytotoxic effects on cancer cells. They stand out for their superior structure, stability, and greater surface area compared to other materials.

The use of silver for wound healing is advantageous due to its effectiveness against multi‐resistant and biofilm‐forming bacteria. Studies show that pure silver nanoparticles can treat inflammation by regulating cytokines and promoting healing with less scar formation (Hamdan et al. 2017). The combination of natural extracts, such as curcumin and açai, with the properties of silver nanoparticles can act in synergy, ensuring greater effectiveness in wound treatment (Gusmão et al. 2023).

Recent reports highlight that the use of plants in the manufacture of nanoparticles offers advantages such as accessibility, safe handling, and a variety of biomolecules known to facilitate the synthesis of these particles (Kumar et al. 2013).

The application of curcumin‐stabilized silver nanoparticles (AgNPs) has been shown to be an effective approach for wound treatment. In fact, silver nitrates have been successfully used in various ophthalmological and dental contexts, in addition to being used in wound healing. According to the study by Maghimaa and Alharbi (Maghimaa and Alharbi 2020), silver nanoparticles synthesized with *C. longa* L. demonstrated considerable potential to accelerate wound healing by promoting the proliferation and migration of fibroblasts. Furthermore, the stabilizers present in the nanoparticles help prevent agglomeration and minimize their cytotoxicity. The biomolecules on the surface of AgNPs can increase their biocompatibility, making them safe for various biomedical applications (Simon et al. 2022).

It is important to highlight that the therapeutic window for the use of AgNPs‐Cur was safe under the tested conditions. Although a reduction in fibroblast viability was observed at higher concentrations (10% and 20%), cell viability remained above 80%, which is considered non‐cytotoxic. Furthermore, the topical application of AgNPs‐Cur in vivo was conducted acutely over 5 days and did not result in any observable damage to mucosal tissues. These findings are consistent with previous studies involving chronic exposure to silver nanoparticles, which also reported no pathological changes in respiratory or nasal tissues (Hyun et al. 2008). In addition, our analyses of oxidative stress, inflammatory cell infiltration, and cytokine expression support the biocompatibility of AgNPs‐Cur, further reinforcing its potential for safe topical use, including on mucosal surfaces.

The antioxidant activity of açaí has also been investigated in some in vivo studies with rats. After 6 weeks of açaí ingestion, a reduction in carbonyl proteins and serum concentrations of total, free, and protein sulfhydryl groups was observed (Pala et al. 2018). Furthermore, the combination of low‐level laser therapy and açaí extract has been reported to have antioxidant, anti‐inflammatory, and antiapoptotic properties, as well as stimulating fibroblast proliferation (Felin et al. 2022).

In this study, we sought to analyze the evolution of the inflammatory process during tissue repair after treatment with silver nanoparticles synthesized in an ecological way. Initially, we observed the pro‐inflammatory cytokines TNF‐α and IL‐1β, which are crucial in the initiation of acute inflammation. However, it is essential that its production is regulated to avoid chronic inflammation, a significant factor in many metabolic diseases and wound healing (Dos Santos et al. 2022). Therefore, identifying compounds that can prevent prolonged inflammation holds promise for treating such conditions and for overall health.

The results obtained show that the treatment of palatal wounds with AgNPs synthesized with curcumin or açaí resulted in a significant reduction in TNF‐α levels. Furthermore, AgNPs‐Cur was effective in decreasing IL‐1β compared to the control group. Similar results were found in the study carried out by Thirupati et al. (Thirupathi et al. 2023) in which GNPs were used in the treatment of palatal wounds in order to minimize the effects of inflammation, highlighting their potential to effectively accelerate tissue repair. Mechanistic studies of the anti‐inflammatory effect of AgNPs demonstrate their ability to inhibit NF‐κB and, consequently, decrease pro‐inflammatory cytokines produced post‐transcriptionally (Abdellatif et al. 2020). With regard to anti‐inflammatory cytokines, only AgNPs‐Cur demonstrated a significant increase in both IL‐4 and TGF‐β compared to the control group. These effects can inhibit the production of pro‐inflammatory cytokines and decrease the activation of NF‐κB. The increase of IL‐4 may also be associated with the activation of TGF‐β, which regulates cell proliferation, differentiation, and morphogenesis in lung tissue during the proliferative phase, accelerating the wound healing process (Saito et al. 2018). Furthermore, based on their anti‐inflammatory properties, studies indicate that AgNPs have the potential to promote the conversion of pro‐inflammatory M1 macrophages into a pro‐repair M2 phenotype, playing a crucial role in the repair mechanism. AgNPs are capable of causing apoptosis of M1 macrophages and reducing ROS so that the phenotype changes to M2 (Yang et al. 2021).

AgNPs synthesized with curcumin were also effective in reducing the levels of the oxidizing markers DCF and nitrite, compared to the control group. AgNPs with açaí also demonstrated a significant reduction of nitrite in palatal wounds. As reported by Chopra and Dey (Chopra et al. 2021), the administration of curcumin, due to its high antioxidant capacity, reduces oxidative stress in the body. The effectiveness in reducing oxidative stress was particularly notable in curcumin nanoparticles, as evidenced in a study carried out by Potphode and Daunde (Potphode et al. 2018).

A crucial aspect of curcumin's antioxidant action against oxidative stress is its ability to increase the activity of superoxide dismutase, glutathione, and catalase, which helps reduce oxidative stress in the mitochondria. Curcumin's ability to penetrate mitochondria protects against oxidative damage and prevents mitochondrial dysfunction. Additionally, curcumin can inhibit stress‐sensitive kinases and scavenge free radicals, which helps prevent significant cell damage (Sathyabhama et al. 2022).

According to Elsilk and Khalil (Elsilk et al. 2022), AgNPs can play a crucial role in minimizing the adverse effects associated with excessive production of nitric oxide (NO) in the body, as they can reduce its synthesis. Additionally, the ability to eliminate NO can prevent negative effects such as increased vascular permeability, protein denaturation, and changes in membranes, all of which contribute to inflammatory processes.

Curcumin, by controlling inflammation, promotes the early transition to the advanced phases of healing. The absence of fibroblast proliferation and migration results in ineffective healing. Thus, the presence of fibroblasts at the site of injury is crucial for efficient healing. Curcumin stimulates fibroblast infiltration into affected areas (Chaushu et al. 2021). Studies with animals treated with curcumin showed accelerated re‐epithelialization, better neovascularization, and increased migration of fibroblasts, myofibroblasts, and macrophages to the wound bed, in addition to an increase in collagen content (Habiboallah et al. 2008).

In this study, a significant decrease in the average number of inflammatory cells and a notable increase in the collagen area were observed in palatal wounds treated with AgNPs‐Cur compared to the control group. There was also a significant increase in the rate of contraction of wounds treated with AgNPs, both curcumin and açaí. Reduced inflammation and accelerated re‐epithelialization contribute to faster healing. A possible factor involved in reducing inflammation in oral wounds is a leukocyte protease secretory inhibitor, an anti‐inflammatory found in the mucosa (Ashcroft et al. 2000).

Kwan and Liu (Kwan et al. 2011) reported that AgNPs are effective in wound healing, showing better collagen alignment after healing compared to control. This effect can be attributed to the ability of AgNPs to regulate collagen deposition and inhibit its disordered growth, promoting adequate alignment and spatial arrangement of the matrix. This is achieved through controlled differentiation of fibroblasts and collagen production.

The results of Liu and Lee (Liu et al. 2010) corroborate that AgNPs promote the proliferation and migration of keratinocytes from the edge to the center of the wound, stimulating cellular maturation and differentiation of fibroblasts into myofibroblasts during healing. Both re‐epithelialization and wound contraction appear to be enhanced by the specific effects of silver on keratinocytes and fibroblasts.

Maghimaa and Alharbi (Maghimaa and Alharbi 2020) demonstrated that AgNPs synthesized with curcumin promote significant migration of fibroblasts, resulting in a considerable improvement in the speed of wound healing. The encapsulation of curcumin in nanoparticles proves to be an advantageous approach for its delivery (Krausz et al. 2015).

Evidence suggests that curcumin plays a positive role in protecting against fibrosis by modulating several molecular pathways, influencing a variety of cytokines, growth factors, and their receptors, as well as inhibiting NF‐κB activation and macrophage infiltration, and negatively regulating inflammatory markers such as TNF‐α, IL‐1, and IL‐6 (Gorabi et al. 2020).

The results of this study are in line with those of Song and Wu (Song et al. 2019), which indicate that AgNPs synthesized with curcumin show better properties due to the synergistic effect between silver nanoparticles and curcumin, resulting in an increase in the release of Ag+ in the presence of curcumin. The large surface area of AgNPs improves contact with microorganisms, providing good antibacterial capacity even at lower concentrations. When AgNPs enter a pathogen, they release silver ions that destroy it. Several mechanisms have been proposed to explain the antibacterial activity of AgNPs, such as the role of surface coating agents, the generation of reactive oxygen species, and the stress caused by Ag+ ions.

The literature has already shown that metal nanoparticles reduced with citrate tend to cause greater chemical toxicity (Rónavári et al. 2021). Therefore, there is a need to study ways to synthesize metallic NPs that reduce their cytotoxic effects. Folhas of *Aloe vera*, Açaí, and curcumina are only some examples of plant extracts that have been used to breed metallic nanoparticles recently (Mendes et al. 2022).The biosynthesis using Açaí and curcumin was proposed based on previous studies employing natural extracts for the synthesis of AgNPs, which demonstrated reduced tissue toxicity along with anti‐inflammatory and antioxidant effects (Alves et al. 2018 Abdellah et al. 2018; Taipe et al. 2024).

In addition to the widely documented antimicrobial effects of AgNPs, this study demonstrates that their use can also significantly contribute to the tissue repair process.

## 5 Conclusions

The topical use of silver nanoparticles reduced with curcumin and açaí extracts, especially AgNPs‐Cur, represents a significant advancement in the treatment of palatal surgical wounds and lesions in the oral mucosa, such as ulcers. This treatment can help mitigate excessive inflammatory responses and promote more efficient wound healing.

However, additional studies are needed to investigate other mechanisms involved in palatal tissue regeneration and to evaluate the effectiveness of these therapies in different experimental models of oral mucosal injuries, as well as their long‐term effects on this tissue. Evaluation of oral microbiology and the effects that AgNPs may have on intestinal microbiology are also topics of future research.

## Author Contributions

**Morgana Francisco Machado Guzzatti:** conceptualization, validation, formal analysis, investigation, data curation, writing. **Ligia Milanez Venturini:** investigation and formal analysis. **Laura de Roch Casagrande:** investigation, validation, formal analysis. **Igor Ramos Lima:** investigation and formal analysis. **Camila da Costa:** investigation and formal analysis. **Ellen de Pieri:** data curation. **Lariani Tamires Witt Tietbohl:** data curation. **Paulo Emilio Feuser:** validation and resources. **Ricardo Andrez Machado de Ávila:** validation and resources. **Anand Thirupathi, Yaodong Gu:** validation and resources. **Paulo Cesar Lock Silveira:** supervision, project administration, funding acquisition. All authors reviewed the manuscript. All authors have read and agreed to the published version of the manuscript.

## Ethics Statement

All animal experiments were conducted following the guidelines of the National Institutes of Health (Bethesda, MD, USA) for the Care and Use of Laboratory Animals and were approved by the Ethics Committee of Universidade do Extremo Sul Catarinense (UNESC) under protocol number 81/2022. Additionally, all procedures adhered to the ARRIVE guidelines (Percie du Sert et al. 2020).

## Consent

The authors have nothing to report.

## Conflicts of Interest

The authors declare no conflicts of interest.

## Data Availability

Data sets analyzed during the current study are available from the corresponding author (Silveira PCL) on reasonable request.
